# Risk of adverse coronavirus disease 2019 outcomes for people living with HIV

**DOI:** 10.1097/QAD.0000000000002836

**Published:** 2021-02-10

**Authors:** Maya M. Mellor, Anne C. Bast, Nicholas R. Jones, Nia W. Roberts, José M. Ordóñez-Mena, Alastair J.M. Reith, Christopher C. Butler, Philippa C. Matthews, Jienchi Dorward

**Affiliations:** a Medical Sciences Division; b Nuffield Department of Primary Care Health Sciences; c Outreach Librarian Knowledge Centre, Bodleian Healthcare Libraries; d Nuffield Department of Medicine, University of Oxford; e Department of Infectious Diseases and Microbiology; f NIHR Biomedical Research Centre, Oxford University Hospitals NHS Foundation Trust, Oxford, UK; g Centre for the AIDS Programme of Research in South Africa, University of KwaZulu-Natal, Durban, South Africa.

**Keywords:** AIDS, antiretroviral therapy, coronavirus disease 2019, HIV, severe acute respiratory syndrome coronavirus 2

## Abstract

**Objective:**

To assess whether people living with HIV (PLWH) are at increased risk of coronavirus disease 2019 (COVID-19) mortality or adverse outcomes, and whether antiretroviral therapy (ART) influences this risk.

**Design:**

Rapid review with meta-analysis and narrative synthesis.

**Methods:**

We searched databases including Embase, Medline, medRxiv and Google Scholar up to 26 August 2020 for studies describing COVID-19 outcomes in PLWH and conducted a meta-analysis of higher quality studies.

**Results:**

We identified 1908 studies and included 19 in the review. In a meta-analysis of five studies, PLWH had a higher risk of COVID-19 mortality [hazard ratio 1.95, 95% confidence interval (CI): 1.62–2.34] compared with people without HIV. Risk of death remained elevated for PLWH in a subgroup analysis of hospitalized cohorts (hazard ratio 1.60, 95% CI: 1.12–2.27) and studies of PLWH across all settings (hazard ratio 2.08, 95% CI: 1.69–2.56). Eight other studies assessed the association between HIV and COVID-19 outcomes, but provided inconclusive, lower quality evidence due to potential confounding and selection bias. There were insufficient data on the effect of CD4^+^ T-cell count and HIV viral load on COVID-19 outcomes. Eleven studies reported COVID-19 outcomes by ART-regimen. In the two largest studies, tenofovir disoproxil fumarate-based regimens were associated with a lower risk of adverse COVID-19 outcomes, although these analyses are susceptible to confounding by co-morbidities.

**Conclusion:**

Emerging evidence suggests a moderately increased risk of COVID-19 mortality among PLWH. Further investigation into the relationship between COVID-19 outcomes and CD4^+^ T-cell count, HIV viral load, ART and the use of tenofovir disoproxil fumarate is warranted.

## Introduction

By September 2020, over 30 million people worldwide had been diagnosed with severe acute respiratory syndrome coronavirus 2 (SARS-CoV-2) [1]. Although SARS-CoV-2 infection may be asymptomatic or cause only mild symptoms, a proportion of people develop severe coronavirus disease 2019 (COVID-19), leading to hospitalization, acute respiratory distress syndrome or death. Established risk factors for severe COVID-19 among the general population include older age [2], chronic kidney disease and obesity [3].

People living with HIV (PLWH), who constitute approximately 0.5% of the global population [4], may have an increased risk of adverse outcomes from COVID-19 as a result of HIV-associated immune dysfunction [5]. There may also be a higher prevalence of co-morbidities among PLWH that predispose to unfavourable COVID-19 outcomes [6]. Conversely, PLWH may have more favourable outcomes due to increased health awareness or close medical follow-up. Some antiretroviral agents are under consideration as potential treatments for COVID-19 [7], but the influence of antiretroviral therapy (ART) on COVID-19 outcomes is not known. In this rapid review, we aim to evaluate the evidence regarding the risk of adverse COVID-19 outcomes in PLWH, and the extent to which this risk is modified by other factors including ART.

## Methods

We used rapid review methods, a simplified version of a systematic review to allow for timely publication [8], to identify studies between 1 January 2020 and 26 August 2020 that described COVID-19 outcomes in PLWH and compared outcomes with HIV-negative people or the general population, or that compared outcomes by risk factors among PLWH. We searched Embase, Medline, medRxiv, LitCovid, Trip, Google and Google Scholar without language restrictions. Search terms are available in Table S1. One author with extensive literature search expertise performed the initial screen to exclude duplicates and studies not related to HIV. For remaining articles, one author performed title and abstract screening, with subsequent full text review by two authors using a standardized data extraction form. In case of disagreement, inclusion decisions were made by a third author. We included preprints to capture emerging evidence. Studies with 15 or less participants were excluded as they were unlikely to be powered to detect meaningful associations. We critically appraised the quality of studies using checklists for Case Series and for Cohort Studies from the Joanna Briggs Institute [9].

Cohort studies reporting COVID-19-related death in people with and without HIV that adjusted for age, sex and co-morbidities were included in a meta-analysis. Cohort-specific relative risks (RRs) and hazard ratios were combined with random effects model to account for variability of the true effect between studies. Hazard ratios and RRs numerically approximate each other with shorter follow-up, rarer endpoints and risks closer to 1 [10]. Subgroup analyses were conducted by study setting and method of confounder adjustment. Meta-analysis was performed in R (version 3.6.0; R Foundation for Statistical Computing, Vienna, Austria) using the *meta* package [11].

## Results

### Summary of included studies

**Fig. 1:**
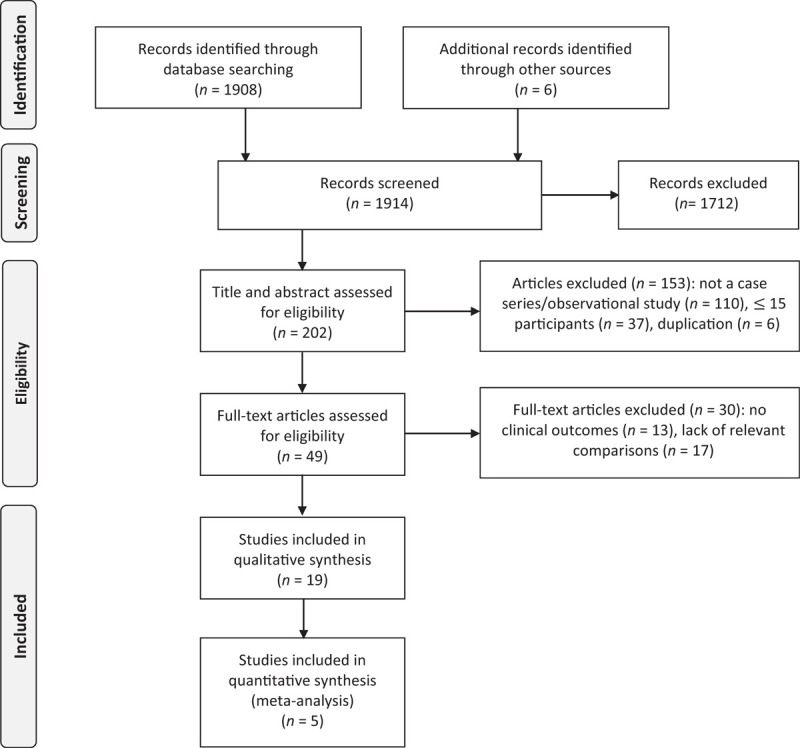
PRISMA flow diagram to show studies identified and included in a systematic meta-analysis of outcomes of coronavirus disease 2019 in people living with HIV.

We identified 1908 records and included 19 studies in our final qualitative analysis (Fig. 1). All included studied were peer-reviewed [12–30]. Quality appraisal is included in Table 1, Tables S1 and S2.

PRISMA, Preferred Reporting Items for Systematic Reviews and Meta-analyses.

**Table 1:** Details of all included studies with summary of mortality findings and quality appraisal.

Study details	Location	Study type and study population	Characteristics of PLWH	Mortality	Quality appraisal^a^
Studies including all COVID-19 patients (hospitalized and in the community)					
Boulle *et al.*[20]	Multi-centre: Western Cape, South Africa	Prospective cohort study of 540 552 PLWH (**3978** confirmed COVID-19) & 2920 380 HIV-negative adults (18 330 confirmed COVID-19)	34% male, median age 20–39 years. No data on proportions by ethnicity, ART, or any co-morbidity. 7.6% with VL > 1000 copies/ml or CD4^+^ cell count <200 cells/μl, 34.7% with VL unknown in past 15 months. No data on corticosteroid use	115 (2.1/10 000) COVID-19 deaths in PLWH vs. 510 (1.7/10 000) in adults without HIV. Adjusted for age and sex, aHR: 1.97; 95% CI: 1.59–2.45. Adjusted for age, sex and co-morbidities: aHR: 2.14; 95% CI: 1.70–2.70^b^	JBI C = 11. Risk of confounding by socio-economic status and BMI
Bhaskaran *et al.*[22]	Multi-centre, United Kingdom	Retrospective population-based cohort analysis of primary care data of 27 480 PLWH and 17 282 905 adults without HIV. Numbers diagnosed with COVID-19 not presented	65% male, median age 40–55 years, 46.2% White, 5.1% Mixed, 4.3% South Asian, 26% Black, 2.3% Other. No data on proportions with any co-morbidity, or by ART, CD4^+^ cell count, viral suppression. No data on systemic corticosteroid use	25 (9.1/10 000) COVID-19 deaths in PLWH vs. 14 857 (8.6/10 000) in adults without HIV. Adjusted for age and sex, aHR: 2.90, 95% CI: 1.96–4.30. Adjusted for age, sex, ethnicity, deprivation and co-morbidities, aHR 2.30; 95% CI: 1.55–3.41^c^	JBI C = 11. Small number of outcomes among PLWH therefore possible overfitting of model
Hadi *et al.*[12]	Multi-centre, United States	Retrospective cohort study among people with SARS-CoV-2 infection comparing 404 PLWH with 49 763 adults without HIV (including matched cohort of **404** adults^d^)	71% male, mean age 48.1, 50% Black or African-American, 34% White, 13% Latinx, 3% Asian. No data on proportions with any co-morbidity. 70% on ART, no data on CD4^+^ cell count or VL. No data on corticosteroid use	20 (4.95%) COVID-19 deaths in PLWH vs. 1585 (3.19%, RR 1.55, 95% CI: 1.01–2.39) in unmatched cohort, and 15 (3.71%, RR 1.33, 95% CI: 0.69–2.57) in matched cohort	JBI C = 10. Unclear how COVID-19 diagnosed. 1 : 1 matching may result in underpowered analysis
del Amo *et al.*[13]	Multi-centre, 60 Spanish HIV clinics	Cohort study of **236** PLWH receiving ART with COVID-19, recruited from 77 590 patients of HIV clinics	75% male, median age 50–59 years. 100% receiving ART. No data on ethnicity, CD4^+^ cell count, viral load or other co-morbidities. 64% hospitalized, 6% in ICU	20 (8%) PLWH died, giving age and sex standardized risk of death of 3.7/10 000 vs. 2.1/10 000 in general Spanish population	JBI C = 7. Comparison group from a different population. Confounding factors not accounted for
Miyashita *et al.*[23]	Multi-centre: New York, US	Cohort study of people with SARS-CoV-2 infection comparing **161** PLWH to 8751 people without HIV	78% male, median age 51–65 years. No data on ethnicity, ART, CD4^+^, VL, or proportion with any co-morbidity	23 (14%) PLWH died vs. 1235 (14%) without HIV (but in age <50 years), PLWH had higher risk of death (RR 4.36, 95% CI 1.43–13.3)	JBI C = 7. Confounding factors not accounted for. Insufficient follow-up time for some patients
Ho *et al.*[24]	Multi-centre, New York, US	Case series of **93** PLWH who presented to ED with positive SARS-CoV-2 RT-PCR test	72% male, median age 58 years, 40.9% Black, 31.2% Hispanic/Latinx. 96% on ART, 84% VL <50 copies/ml, median CD4^+^ cell count 554 cells/μl	19 (20.4%) PLWH died, no mortality comparison given	JBI CS = 8. Deaths among those not admitted not recorded
Di Biagio *et al.*[25]	Multi-centre, Italy	Case series of **69** PLWH who were diagnoses with SARS-CoV-2 infection	72% male, median age 50–55, 86% white, 100% on ART, 88% VL < 50 copies/ml, median CD4^+^ cell count 580–600 cells/μl	7 (10.8% of those with known outcomes) PLWH died, no mortality comparison given	JBI CS = 5. Not clear if participating centres identified SARS-CoV-2 cases systematically. Statistics unclear
Maggiolo *et al.*[26]	Single-centre, Bergamo, Italy	Cohort study of **55** PLWH with suspected/confirmed COVID-19 vs. 69 asymptomatic PLWH who tested negative for SARS-CoV-2 (RT-PCR or serology)	80% male, median age 54 (IQR: 49–58), 100% receiving ART, 98% VL < 50 copies/ml, median last CD4^+^ cell count 904 cells/μl, 51% with any co-morbidity	4 (7.2%) PLWH died, no mortality comparison given	JBI C = 6. Follow-up unclear. Confounding factors not accounted for. Sample size not large enough for multi-variable analysis
Etienne *et al.*[27]	Single centre, Paris, France	Case series of **54** PLWH with symptoms or were hospitalized with COVID-19	61.1% male, median age 54, 100% on ART, 96.2% VL < 40 copies/ml, median CD4^+^ cell count 583 cells/μl, 44.6% sub-Saharan African origin	1 (1.9%) PLWH died, no mortality comparison given	JBI CS = 3. COVID-19 case definition and disease severity not defined. Short follow-up time
Inciarte *et al.*[28]	Single centre, Barcelona, Spain	Cohort study of 5683 PLWH in of whom **53** PLWH had confirmed or suspected COVID-19	85% male, median age 44, median last CD4^+^ cell count 618 cells/μl (IQR: 449–834), 96% on ART	2 (4%) PLWH died, no mortality comparison given	JBI C = 7. COVID-19 case definition unclear. Duration of follow-up unclear. Confounding not accounted for
Gervasoni *et al.*[29]	Single centre: Milan, Italy	Case series of **47** PLWH with suspected/confirmed COVID-19	74% male, median age 52 years, no ethnicity data. 100% receiving ART, median CD4^+^ cell count 636 cells/μl, 93.6% with undetectable VL. 64% with ≥1 co-morbidity	2 PLWH died (4.2% of cohort, 15.4% of those hospitalized with COVID-19), vs. 17% died among 502 HIV-negative patients admitted with COVID-19 at same hospital	JBI CS = 7. Comparison group from a different population. Length of follow-up unclear. Confounding factors not accounted for
Huang *et al.*[30]	Multi-centre, Wuhan City, China	Cohort study 6001 PLWH of whom **35** were diagnosed with COVID-19	90% male, median age 37 years, no data on ethnicity. 92% on ART, median CD4^+^ cell count 200–499 cells/μl, 66% VL < 20 copies/ml	2 (5.7%) PLWH with COVID-19 died vs. 3869/50 333 (7.69%) of general population of Wuhan with COVID-19	JBI C = 5. Comparison group from a different population. Follow-up not clear. Confounding not accounted for
Härter *et al.*[14]	Multi-centre: 12 German HIV centres	Case series of **33** PLWH with confirmed COVID-19	91% male, median age 48 years, no ethnicity data. 100% receiving ART, overall median CD4^+^ cell count 670 cells/μl, 94% virally suppressed, 60%. with ≥1 co-morbidity	3 PLWH died (9.1%) vs. 3.7% mortality in general COVID-19 positive population in Germany	JBI CS = 7. Comparison group from a different population and confounding not accounted for. Not all patients with an outcome
Studies including only hospitalized COVID-19 patients					
Geretti *et al.*[21]	Multi-centre: 207 UK hospitals	Prospective cohort study of people hospitalized with suspected/confirmed COVID-19. **122** PLWH vs. 47 470 HIV-negative adults	66% male, median age 56 (IQR: 49–62) years. White (45.5%), Black (42.9%), Asian (0.9%), Other (10.7%). PLWH had fewer co-morbidities overall, 74.6% ≥ 1 co-morbidity. 91.8% had a record ART, No data on proportions by CD4^+^ or VL. No data on corticosteroid use	By day 28, 30 PLWH died (24.6%) vs. 13 969 (29.4%) in adults without HIV. Adjusted for age and sex, aHR: 1.45; 95% CI: 1.00–2.12 (*P* = 0.05). Adjusted for age, sex and co-morbidities, aHR: 1.69; 95% CI: 1.15–2.48, *P* = 0.008^e^	JBI C = 11. Risk of confounding by socioeconomic status
Sigel *et al.*[15]	Multi-centre: 5 hospitals New York, US	Cohort study of **88** PLWH hospitalized with laboratory confirmed COVID-19 compared with matched cohort of 405 hospitalized HIV-negative adults^f^	78% male, median age 45–67 years. Ethnicity: White (19%), Black, (40%), Hispanic (30%), Other (11%). No data on proportions with any co-morbidity. 100% on ART, 58% CD4^+^ cell count >200 cells/μl, 81% VL < 50 copies/μl. No data on corticosteroid use	18 (21%) COVID-19 deaths in PLWH vs. 81 (20%) in adults without HIV	JBI C = 6. Follow-up not complete for all patients. Matched on limited number of confounders. Potential overadjustment
Vizcarra *et al.*[16]	Single centre: Madrid	Case series of **51** PLWH with suspected/confirmed COVID-19	84% male mean age 53.3 years, 88% white ethnicity. 100% receiving ART, median CD4^+^ cell count 565 cells/μl, 98% virally suppressed, 63% with ≥1 co-morbidity. 15 (38%) received corticosteroids	2 PLWH died (4%) vs. 20% among general population admitted to nearby hospital with COVID-19	JBI CS = 7. Comparison group from different population. COVID-19 case definition unclear. Not all cases completed follow-up
Shalev *et al.*[17]	Single centre: New York, US	Case series of **31** PLWH hospitalized for COVID-19 at a large tertiary care medical centre in New York City	77% male, mean age 60.7 years, 52% black, 29% Hispanic, 9% white. 100% receiving ART, median CD4^+^ cell count 396 cells/μl, 100% virally suppressed, 71% with ≥1 co-morbidity. 8 (25.8%) received corticosteroids	8 PLWH died (27.6%), no mortality comparison given	JBI CS = 8. Not all cases completed follow-up
Karmen-Tuohy *et al.*[18]	Multi-centre: 4 hospitals in New York, US	Cohort study of **21** PLWH hospitalized with confirmed COVID-19 compared with a matched cohort^g^ of 42 HIV-negative patients (selected from 2617 non-HIV patients with COVID-19 at same centres)	90.5% male, mean age 60 years, 24% African American, 38% White, 38% Other. 100% receiving ART, median CD4^+^ cell count 298 cells/μl, 71% VL < 50 copies/ml. 4 (19%) PLWH received corticosteroids vs. 0 in the HIV-negative matched cohort	6 PLWH died (28.6%) vs. 10 (23.8%) in HIV-negative cohort (*P* = 0.682)	JBI C = 11
Childs *et al.*[19]	Single centre: London, UK	Case series of **18** PLWH hospitalized with confirmed COVID-19	67% male, median age 52, 94% black ethnicity. 100% on ART, median CD4^+^ cell count 395 cells/μl, 94% virally suppressed. No data on corticosteroid use	5 PLWH died (29%), no mortality comparison given	JBI CS = 6. Time period of enrolment and follow-up time not defined

aHR, adjusted hazard ratio; ART, antiretroviral therapy; CI, confidence interval; COVID-19, coronavirus disease 2019; ED, emergency department; IQR, interquartile range; PLWH, people living with HIV; RR, risk ratio; RT-PCR, real-time-polymerase chain reaction; SARS-CoV-2, severe acute respiratory syndrome coronavirus 2; VL, viral load. Bold indicates number of PLWH diagnosed with COVID.

a Joanna Briggs Institute (JBI) Cohort Study (C) or Case Series (CS) checklist items completed, with comment. See Tables S2 and S3 for full scores.

b Adjusted for age, sex, diabetes, tuberculosis history and other co-morbidities (hypertension, kidney disease, lung disease).

c Adjusted for age, sex, deprivation, ethnicity, obesity, smoking and presence of co-morbidities (hypertension, chronic respiratory disease, chronic cardiac disease, diabetes, non-haematological cancer, haematological cancer, chronic liver disease, stroke, dementia, other neurological disease, reduced kidney function, organ transplant, asplenia, rheumatoid arthritis, lupus, psoriasis or other immunosuppresive disorders).

d Matched on age, sex, ethnicity, smoking, BMI, diabetes, hypertension and chronic lung diseases.

e Adjusted for age, sex, ethnicity, baseline date, indeterminate/probable hospital acquisition of COVID-19, 10 co-morbidities and hypoxia/receiving oxygen at presentation.

f Matched on age, sex, race/ethnicity and week of SARS-CoV-2 infection.

g Matched on age, sex, BMI, smoking, co-morbidities (chronic kidney disease, hypertension, asthma, chronic obstructive pulmonary disease, heart failure) and admission date, all from same centre.

We identified five cohort studies (two prospective, three retrospective) comparing COVID-19-related mortality between PLWH and HIV-negative people, which we pooled in a meta-analysis [12,18,20–22]. Four of these reported all-cause mortality among people diagnosed with COVID-19 [12,18,20,21] and one reported mortality due to COVID-19 as recorded on death certificates [22]. Of the remaining 14 studies, seven made multiple comparisons between PLWH with COVID-19 and HIV-negative cohorts and/or the general population, and/or PLWH without COVID-19, [13,14,16,17,27,29,30], two studies compared only to a HIV-negative cohort [15,23], two studies compared only cohorts of PLWH with and without COVID-19 [19,26] and three studies compared only the characteristics of PLWH with different COVID-19 disease severity [24,25,28].

There were 10 studies that included a total of more than 1000 individuals, but among these the median number of PLWH with COVID-19 co-infection was only 55 (interquartile range 35–115). Seventeen of the studies were performed in high-income countries and most included a majority of patients on ART with well controlled HIV (Table 1).

### Quality of evidence and risk of bias assessment

There were common limitations among the included studies (Tables S2 and S3). Most were retrospective analyses of routinely collected clinical data, meaning identification of COVID-19 cases was not systematic and depended on the local approach to screening and diagnosis. This has varied over time and between settings, and may also differ between PLWH and the general population. Only five studies directly compared COVID-19 outcomes among PLWH and HIV-negative people in the same cohort, and accounted adequately for potential confounding by co-morbidities associated with adverse COVID-19 outcomes. Other studies used inadequately matched HIV-negative controls, or general populations in various settings, which is susceptible to bias as the exposed and control groups were selected differently [2]. Across all studies, the numbers of PLWH and COVID-19 infection were relatively low.

### Adjusted analyses of HIV and risk of death in coronavirus disease 2019

**Fig. 2:**
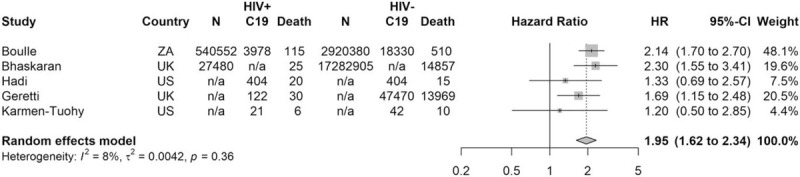
Meta-analysis of the effect of HIV on risk of coronavirus disease 2019 death.

In a meta-analysis of five cohort studies which accounted for confounding, the risk of death from COVID-19 for PLWH was almost double that of HIV-negative people [hazard ratio = 1.95, 95% confidence interval (CI) 1.62–2.34] (Fig. 2) [12,18,20–22]. Three of these studies used large routine databases to identify PLWH across community and hospital settings, in South Africa [20], the United Kingdom [22] and United States [12], and two studies were limited to hospitalized PLWH and COVID-19 in the United Kingdom [21] and United States [18]. In a subgroup analysis there was no significant difference between study settings (*P* = 0.20), although a weaker hazard ratio was seen in hospitalized patients (Fig. S1). Among the three studies which used multi-variable adjustment to account for confounding [20–22], the *crude* risk of COVID-19 death was similar between people with and without HIV, but after adjustment for age, the adjusted risk among PLWH was higher. Subsequent adjustment for co-morbidities did not drastically alter hazard ratios (Table 1). In subgroup analysis by method of accounting for confounders, a weaker hazard ratio was seen in the smaller two studies which used propensity score matching (Fig. S2) [12,18].

Country; UK, United Kingdom; US, United States; ZA, South Africa. HIV+ and HIV− refers to people with and without HIV. C19 refers to those with coronavirus disease 2019. The denominators (*N*) refer to the original population where reported: A cohort of people diagnosed with HIV and the wider general population without HIV. Not all studies reported this information. Results are reported as hazard ratios with 95% confidence intervals.

### Adjusted analyses of risk of hospitalization and morbidity in people living with HIV

Three of the five cohort studies conducted analyses of the association between HIV status and the risk of other COVID-19 outcomes [12,18,21]. Among 47 592 individuals hospitalized with COVID-19 in the United Kingdom, the odds of critical care admission was higher among the 122 PLWH [odds ratio (OR) 2.79, 95% CI 1.90–4.08], but this was attenuated after adjustment for demographics and co-morbidities (OR 1.22; 95% CI 0.80–1.87; *P* = 0.35) [21]. In the analysis of 50 167 people with COVID-19 in the United States, 404 PLWH (*n* = 404) were at increased risk of hospitalization compared with 1 : 1 propensity score matched HIV-negative controls (RR 1.70, 95% CI: 1.21–2.38) [12]. Lastly, among 2638 people hospitalized with COVID-19 in the United States, there were six (28.6%) ICU admissions among 21 PLWH, compared with seven (16.7%) among a propensity score matched cohort of 42 HIV-negative people (*P* = 0.271) [18].

### Unadjusted analyses of coronavirus disease 2019-related outcomes in people living with HIV

Eight studies provided lower quality evidence regarding COVID-19 outcomes in PLWH as they did not compare HIV-positive and negative people in the same cohort, or did not adequately account for confounders [13–17,23,29,30] (Table 1, Tables S2 and S3). The largest of these was a Spanish multi-centre study of 77 590 PLWH, of whom 236 were diagnosed with COVID-19 and 20 died. In keeping with our meta-analysis result, age-standardized and sex-standardized mortality from COVID-19 were found to be higher in PLWH (3.7 per 10 000) compared with the general population (2.1 per 10 000) [13]. The other seven studies [14–17,23,29,30] were limited due to being at single sites, having small sample sizes (median 64, range *n* = 31–161 PLWH diagnosed with COVID-19) and not accounting for potential confounding. These studies report conflicting results with one suggesting a higher rate of hospitalization and mortality among PLWH compared with the general COVID-19-positive population [14], two studies suggesting lower COVID-19 mortality in PLWH [16,29] and four studies reporting no significant difference in the risk of adverse outcomes from COVID-19 between PLWH and HIV-negative cohorts [15,23] or the general population [17,30].

### Risk of death and hospitalization in relation to CD4^+^ T-cell count and HIV viral load

Several of the large cohort studies did not include data on CD4^+^ T-cell count or HIV viral loads [12,21,22]. In the South African study, lower CD4^+^ T-cell counts (measured during the COVID-19 episode) were associated with mortality, but this could be a result of, rather than causing, severe disease (Table 2). There was no difference in outcomes by HIV viral load, although viral load data were incomplete and numbers with unsuppressed viral loads were small [20]. A London HIV clinic found that 18 PLWH who were hospitalized with COVID-19 had a lower median CD4^+^ T-cell count (395 vs. 573 cells/μl, *P* = 0.03) compared with their 2699 PLWH outpatients (Table 2) [19]. A further nine studies (median *n* = 54 PLWH and COVID-19, range *n* = 35–93) found no significant association between CD4^+^ T-cell count or HIV viral load and COVID-19 outcomes [15,16,18,24–28,30].

**Table 2:** Summary of studies reporting outcomes by antiretroviral therapy regimen and other risk factors for adverse coronavirus disease 2019 outcomes among people living with HIV.

Study	Influence of ART regimens	Other risk factors among PLWH
Boulle *et al.*[20], *n* = 3978	Lower mortality in patients on TDF vs. abacavir/zidovudine (aHR: 0.42; 95% CI: 0.22; 0.78)	601 patients had CD4^+^ cell count measured during episode of COVID-19. Higher mortality associated with CD4^+^ cell counts <200 cells/μl (*n* = 70) vs. ≥350 cells/μl (aHR 1.97; 95% CI 1.14–3.40). Direction of causality unclearNo difference in hazard of COVID-19 death by HIV VL (aHR vs. HIV-negative: 2.61 (95% CI: 1.98–3.43) for VL < 1000 copies/ml; 3.35 (95% CI: 1.38–6.12) for VL ≥ 1000 copies/ml or CD4^+^ cell count <200 cells/μl)
Bhaskaran *et al.*[22]Number of PLWH & COVID-19 not presented	N/A	PLWH of Black ethnicity had higher risk of COVID-19 mortality (aHR 4.31, *P* for interaction 0.044). No data on outcomes by CD4^+^ or VL
del Amo *et al.*[13], *n* = 236	Lowest risk for COVID-19 diagnosis [16.9% (95% CI: 10.5–25.9) & hospitalization 10.5 (95% CI: 5.6–17.9)] in PLWH receiving TDF/FTC compared with other ART regimens [e.g. ABC/3TC 28.3% (95% CI: 21.5–36.7) and 23.4% (95% CI: 17.2–31.1) respectively]	Higher crude risk of COVID-19 death among older PLWH [70–79 years = 26.6/10 000, 95% (CI: 10.7–54.9) vs. 50–59 years 2.2/10 000 (95% CI: 10.7–54.9)]. No difference by sex
Ho *et al.*[24], *n* = 93	No significant difference in TDF use between PLWH with COVID-19 who survived and died (73.6 vs. 55.5%, *P* = 0.15)	No significant differences in obesity, CD4^+^ cell counts or HIV VL between PLWH with COVID-19 who survived and died
Di Biagio *et al.*[25], *n* = 69	No stat. sig. association between risk of hospitalization and ART regimens	Hospitalized PLWH were slightly older (*P* = 0.047). No association between most recent VL or CD4^+^ cell count and hospitalization
Maggiolo *et al.*[26], *n* = 55	No difference in TDF use among PLWH with COVID-19 (60%) vs. without COVID-19 (60.8%)	4 PLWH with COVID-19 who died had lower last CD4^+^ cell count (median 514 cells/μl) than the 51 PLWH who survived (median 913 cells/μl)
Etienne *et al.*[27], *n* = 54	No stat. sig. difference between ART regimen and COVID-19 severity	No stat. sig. association between CD4^+^ cell counts or VL < 40 copies/ml and COVID-19 severity
Inciarte *et al.*[28], *n* = 53	No associations between ART regimen and COVID-19 severity	No association between latest CD4^+^ cell count and COVID-19 severity
Huang *et al.*[30], *n* = 35	N/A	Older age and ART discontinuation associated with COVID-19 infection. No association between latest CD4^+^ cell count or VL and COVID-19 infection
Geretti *et al.*[21], *n* = 115	N/A	Age, obesity and diabetes were associated with COVID-19 death among PLWH. No data on CD4^+^ cell counts or viral loads. 25/30 PLWH who died (80.7%) had an ART record, compared with 87/92 (94.6%) of those who survived (*P* = 0.07)
Sigel *et al.*[15], *n* = 88	PLWH who survived were more likely to have been treated with NRTIs than those PLWH who died (99 vs. 89%, *P* = 0.04) in univariate analysis. No difference in outcomes for other classes of ART	No association between co-morbidities, latest CD4^+^ cell count or VL and COVID-19 death
Vizcarra *et al.*[16], *n* = 51	Increased TAF use in PLWH with COVID-19 (37/51, 73%), vs. PLWH without COVID-19 (38%, *P* = 0.0036)	PLWH with COVID-19 were significantly more likely to have co-morbidities (63 vs. 38%, *P* = 0.00059), and had higher median BMI (25.5 vs. 23.7 kg/m^2^, *P* = 0.021) compared with 1288 PLWH without COVID-19. No association between CD4^+^ T cell count and SARS-CoV-2 infection or adverse COVID-19 outcomes
Shalev *et al.*[17], *n* = 31	7/8 (88%) PLWH who died from COVID-19 used TAF/TDF vs. 10/23 (43%) of those who survived	N/A
Karmen-Tuohy *et al.*[18], *n* = 21	N/A	No association between most recent CD4^+^ cell count and mortality (OR 0.996, 95% CI: 0.992–1.11)
Childs *et al.*[19], *n* = 18	More common use of protease inhibitor–containing ART regimens among PLWH with COVID-19 (OR, 2.43, 95% CI, 0.94–6.29)	PLWH hospitalized with COVID-19 were more likely to be of black ethnicity (OR: 12.22, 95% CI: 1.62–92.00), and had lower median CD4^+^ cell counts (395 vs. 573, *P* = 0.03)

3TC, lamivudine; ABC, abacavir; aHR, adjusted hazard ratio; ART, antiretroviral therapy; CI, confidence interval; COVID-19, coronavirus disease 2019; FTC, emtricitabine; NRTIs, nucleotide reverse transcriptase inhibitors; OR, odds ratio; PLWH, people living with HIV; SARS-CoV-2, severe acute respiratory syndrome coronavirus 2; TAF, tenofovir alafenamide; TDF, tenofovir disoproxil fumarate; VL, viral load.

### Impact of antiretroviral therapy regimen on coronavirus disease 2019 outcomes

One study compared COVID-19 outcomes between PLWH receiving and not receiving ART. In an unadjusted analysis among PLWH hospitalized with COVID-19, 25/30 who died (80.7%) had an ART record, compared with 87/92 (94.6%) of those who survived (*P* = 0.07) [21]. We identified 11 studies assessing the relationship between specific ART regimens and COVID-19 outcomes in PLWH. In South Africa, COVID-19-related mortality was lower in patients on tenofovir disoproxil fumarate (TDF)-based regimens vs. abacavir/zidovudine-based regimens, which are used for patients with co-morbidities or requiring second line treatment [adjusted hazard ratio (aHR): 0.42; 95% CI: 0.22–0.78] [20]. While this analysis was adjusted for certain co-morbidities, the observed association may be confounded due to patients receiving TDF having less complex healthcare needs. In the Spanish multi-centre study, PLWH receiving TDF and emtricitabine had the lowest risk for COVID-19 diagnosis (16.9 per 10 000) and hospitalization (10.5 per 10 000) compared with all other ART regimens investigated, but without adjusting for co-morbidities [13]. A US hospital study found that PLWH and COVID-19 who survived were more likely to have been treated with nucleotide reverse transcriptase inhibitors (NRTIs) than those PLWH who died (99 vs. 89%, *P* = 0.04) in univariate analysis [15]. Seven smaller studies (*n* = 18–93 PLWH with COVID-19) reported no significant association between ART-regimen and COVID-19 severity among PLWH [16,17,19,24–28].

### Other factors influencing coronavirus disease 2019 outcomes among people living with HIV

Thirteen studies assessed the influence of co-morbidities and demographics on the outcomes of COVID-19 among PLWH [15,16,18–22,24–28,30]. In the United Kingdom-based cohort study of hospitalized patients, among 122 PLWH with COVID-19, the 30 who died were more likely to have obesity and diabetes [21]. Bhaskaran *et al.* and Childs *et al.* report evidence of a higher risk of COVID-19 death and hospitalization respectively among PLWH of black ethnicity (Bhaskaran *et al.* mortality aHR = 3.80; 95% CI: 2.15–6.74, *P* for interaction = 0.045; Childs *et al.* hospitalization crude OR: 12.22, 95% CI: 1.62–92.00) [19,22]. Other smaller analyses suggested that among PLWH, factors such as older age [27], metabolic disorders [27], obesity [16], African ethnicity [27] and organ transplantation [15] were associated with COVID-19 infection or severity.

## Discussion

### Summary

Emerging evidence suggests an increased risk of COVID-19-related death in PLWH. Whether this increased risk is associated with to HIV viral load, CD4^+^ T-cell counts or ART use was not clear as data in the included studies was insufficient. Regarding differences in effects of specific ART regimens, we found some evidence that TDF-based regimens may be associated with lower frequency of SARS-CoV-2 infection and milder courses of COVID-19 compared with other ART regimens, although this was not consistent between studies and was susceptible to confounding. Risk factors for severe COVID-19 among PLWH include older age, obesity and black ethnicity, and appear similar to the general population.

### Risk of coronavirus disease 2019-related mortality among people living with HIV

In our review, the two population-based studies from South Africa and the United Kingdom both suggested almost double the risk of COVID-19-related death among PLWH, despite having very different demographic profiles [20,22]. In contrast, studies restricted to cohorts of PLWH diagnosed with COVID-19 [12], and hospitalized patients with COVID-19 [18,21] found a weaker or null effect. These studies are more at risk of selection bias, as PLWH with milder symptoms may be more likely to test for SARS-CoV-2 or be hospitalized by clinicians (due to a higher perceived risk), compared with people without HIV who may only be tested or hospitalized once more severely unwell. This would lead to the cohort of PLWH being less unwell at baseline compared with the HIV-negative cohort, leading to underestimation of any association between HIV status and COVID-19-related mortality. Furthermore, studies restricted to hospitalized patients cannot account for the effect of HIV (or any other potential risk factor) on SARS-CoV-2 infection and COVID-19 severity which result in hospitalization, and therefore may underestimate the effect of risk factors on COVID-19 death, compared with studies in the general population [31].

### Influence of antiretroviral therapy

We found no evidence to determine whether ART use reduces COVID-19 severity through immune reconstitution, as most studies only included PLWH on ART. Regarding specific antiretrovirals, the potential therapeutic value of TDF for COVID-19 is supported by results from molecular docking studies [32]. However, TDF is relatively contra-indicated in renal impairment [33], meaning patients receiving TDF-based ART are likely to have less co-morbidities, which may explain the observed better COVID-19 outcomes. Randomized trials of TDF prophylaxis for SARS-CoV-2 are underway [34].

### Comparisons with existing literature

PLWH are known to be at higher risk of respiratory bacterial infections, but the evidence regarding acute viral infections is less clear [35]. A review from the H1N1 influenza pandemic in 2009/2010 found some evidence of a higher risk of adverse H1N1 outcomes among PLWH who were severely immunocompromised [36]. However, the quality of the evidence was weak with a lack of rigorously designed prospective cohort studies, reflecting the challenges of in-pandemic research [36].

As of 26 August 2020, we identified seven systematic reviews on COVID-19 in PLWH [37–43]. All these reviews lacked the more robust evidence from the recent large cohort studies that this review addresses [20–22]. Moreover, one review included articles assessing non-HIV-related immunodeficiency [40] and four did not address the influence of ART [37,41–43].

### Limitations

Our meta-analysis of five studies is potentially limited by the small numbers of PLWH with COVID-19 who died. This presented challenges when accounting for confounding; studies that used multi-variable analyses to adjust for confounding were susceptible to overfitting of models and potential overadjustment by factors which could be on the causal pathway between HIV and death (e.g. malignancy or tuberculosis). Studies that used matching were potentially underpowered, which may explain why they tended to report no independent association between HIV and COVID-19 death. Concomitant treatment with corticosteroids, which reduce COVID-19 mortality [44] and may have been used differently by HIV status, was only reported by one study [18]. In our narrative synthesis, the majority of the studies were small case series or cohort studies that did not adequately account for confounders such as age. Most were performed in high-income countries, and the majority of participants had well controlled HIV on ART. This may limit the applicability to populations of PLWH in other settings. Only 68% of adults and 53% of children living with HIV globally are receiving ART [4], highlighting a crucial need to examine the risk of COVID-19 complications in these populations.

### Conclusion

We present evidence which suggests a moderately increased risk of COVID-19 death among PLWH. Measures to mitigate COVID-19 risk among PLWH should be included in HIV programs. Further research into the role of ART, immunosuppression and viral suppression is needed to quantify and address risks for PLWH in diverse settings.

## Acknowledgements

Author contributions: M.M.M., N.R.J. and J.D. conceived the study. N.W.R. performed the literature search. M.M.M. and A.C.B. contributed equally to the screening and qualitative analysis, with support from N.R.J. and J.D. J.M.O.-M. performed the meta-analysis. M.M.M. and A.C.B. wrote the first draft of the article. M.M.M., A.C.B., N.W.R., J.M.O.-M., A.J.M.R., C.C.B., P.C.M., N.R.J. and J.D. critically reviewed and edited the article and consented to publication.

N.R.J. and J.D. are funded by the Wellcome Trust PhD Programme for Primary Care Clinicians (216421/Z/19/Z). P.C.M. is funded by a Wellcome Trust Intermediate Fellowship (110110Z/15/Z).

### Conflicts of interest

There are no conflicts of interest.

## Supplementary Material

Supplemental Digital Content
