# Supplementary material for: Risk of adverse coronavirus disease 2019 outcomes for people living with HIV
Source: AIDS. 2021 Feb 10;35(4):F1–F10. doi: 10.1097/QAD.0000000000002836 (PMC7924978; doi:10.1097/QAD.0000000000002836)
Supplement: Supplemental Digital Content [file aids-35-0f1-s001.docx]

|  | MEDLINE (OvidSP) |  |
| --- | --- | --- |
|  | Searches | Results |
| 1 | Coronavirus infections/ | 23644 |
| 2 | (coronavirus or COVID-19 or 2019nCoV or 2019-nCoV or WN-CoV or nCoV or SARS-CoV-2 or HCoV-19).ti,ab,kw. | 58091 |
| 3 | 1 or 2 | 60004 |
| 4 | exp HIV/ or exp HIV Infections/ | 316816 |
| 5 | Antiretroviral Therapy, Highly Active/ | 21328 |
| 6 | (haart or art or anti-retroviral* or antiretroviral*).ti,ab,kw. | 157937 |
| 7 | (hiv or human immunodeficiency virus).ti,ab,kw. | 332463 |
| 8 | 4 or 5 or 6 or 7 | 494710 |
| 9 | 3 and 8 | 845 |
| 10 | exp animals/ not humans/ | 4728498 |
| 11 | 9 not 10 | 830 |
| 12 | limit 11 to yr="2020 -Current" | 620 |
|  |  |  |
|  | **EMBASE (OvidSP)** |  |
|  | Searches | Results |
| 1 | Coronavirus infection/ | 8488 |
| 2 | (coronavirus or COVID-19 or 2019nCoV or 2019-nCoV or WN-CoV or nCoV or SARS-CoV-2 or HCoV-19).ti,ab,kw. | 55744 |
| 3 | 1 or 2 | 56652 |
| 4 | exp Human immunodeficiency virus/ or exp Human immunodeficiency virus infection/ | 483088 |
| 5 | highly active antiretroviral therapy/ or *antiretroviral therapy/ | 42083 |
| 6 | (haart or art or anti-retroviral* or antiretroviral*).ti,ab,kw. | 203774 |
| 7 | (hiv or human immunodeficiency virus).ti,ab,kw. | 424017 |
| 8 | 4 or 5 or 6 or 7 | 659987 |
| 9 | 3 and 8 | 1047 |
| 10 | (exp animals/ or nonhuman/) not human/ | 6566193 |
| 11 | 9 not 10 | 949 |
| 12 | limit 11 to yr="2020 -Current" | 696 |

**Table S1: Search terms**

Note: Similar terms were used in medRxiv ([www.medrxiv.org/](http://www.medrxiv.org/)), LitCovid ([www.ncbi.nlm.nih.gov/research/coronavirus/](http://www.ncbi.nlm.nih.gov/research/coronavirus/)) and TRIPdatabase ([www.tripdatabase.com/](http://www.tripdatabase.com/)). For pre-prints identified in the search which were subsequently published, we used the published, peer-reviewed version.

**Table S1: Quality appraisal of included cohort studies using Joanna Briggs Institute Cohort Studies Checklist**

|  | Davies | Bhaskaran | Hadi YB | Del Amo | Miyashita | Maggiolo | Inciarte A | Huang J | Geretti | Sigel, K | Karmen-Tuohy |
| --- | --- | --- | --- | --- | --- | --- | --- | --- | --- | --- | --- |
| Were the two groups similar and recruited from the same population? | Y | Y | Y | N | Y | Y | Y | N | Y | Y | Y |
| Were the exposures measured similarly to assign people to both exposed and unexposed groups? | Y | Y | Y | N | Y | Y | Y | Y | Y | Y | Y |
| Was the exposure measured in a valid and reliable way? | Y | Y | Y | Y | Y | Y | Y | Y | Y | Y | Y |
| Were confounding factors identified? | Y | Y | Y | N | N | Y | Y | Y | Y | Y | Y |
| Were strategies to deal with confounding factors stated? | Y | Y | Y | N | N | N | N | N | Y | ? | Y |
| Were the groups/participants free of the outcome at the start of the study (or at the moment of exposure)? | Y | Y | Y | Y | Y | Y | Y | Y | Y | Y | Y |
| Were the outcomes measured in a valid and reliable way? | Y | Y | Y | Y | Y | Y | Y | Y | Y | Y | Y |
| Was the follow up time reported and sufficient to be long enough for outcomes to occur? | Y | Y | Y | Y | Y | N | ? | ? | Y | ? | Y |
| Was follow up complete, and if not, were the reasons to loss to follow up described and explored? | Y | Y | Y | Y | ? | ? | Y | ? | Y | N | Y |
| Were strategies to address incomplete follow up utilized? | Y | Y | N | N/A | ? | ? | N/A | ? | Y | N | N/A |
| Was appropriate statistical analysis used? | Y | Y | Y | Y | Y | ? | ? | N | Y | N | Y |
| Score (Y or N/A = 1, N or ? = 0) | 11 | 11 | 10 | 7 | 7 | 6 | 7 | 5 | 11 | 6 | 11 |

**Table S2: Quality appraisal of included case series using Joanna Briggs Institute Case Series Checklist**

|  | Ho | Di Biagio | Etienne, N | Gervasoni | Harter | Vizcarra | Shalev | Childs |
| --- | --- | --- | --- | --- | --- | --- | --- | --- |
| Were there clear criteria for inclusion in the case series? | Y | Y | N | Y | Y | Y | Y | N |
| Was the condition measured in a standard, reliable way for all participants included in the case series? | Y | Y | N | N | Y | N | Y | Y |
| Were valid methods used for identification of the condition for all participants included in the case series? | Y | N | N | N | N | N | Y | Y |
| Did the case series have consecutive inclusion of participants? | Y | N | Y | Y | Y | Y | Y | N |
| Did the case series have complete inclusion of participants? | ? | ? | ? | Y | Y | Y | Y | ? |
| Was there clear reporting of the demographics of the participants in the study? | Y | Y | Y | Y | Y | Y | Y | Y |
| Was there clear reporting of clinical information of the participants? | Y | Y | Y | Y | Y | Y | Y | Y |
| Were the outcomes or follow up results of cases clearly reported? | Y | Y | N | Y | N | ? | N | Y |
| Was there clear reporting of the presenting site(s)/clinic(s) demographic information? | N | N | N | N | N | Y | N | N |
| Was statistical analysis appropriate? | Y | N | ? | Y | Y | Y | Y | Y |
| Score (Y or N/A = 1, N or ? = 0) | 8 | 5 | 3 | 7 | 7 | 8 | 8 | 6 |

# Figure S1: Meta-analysis of the effect of HIV on risk of COVID-19 death, by study population


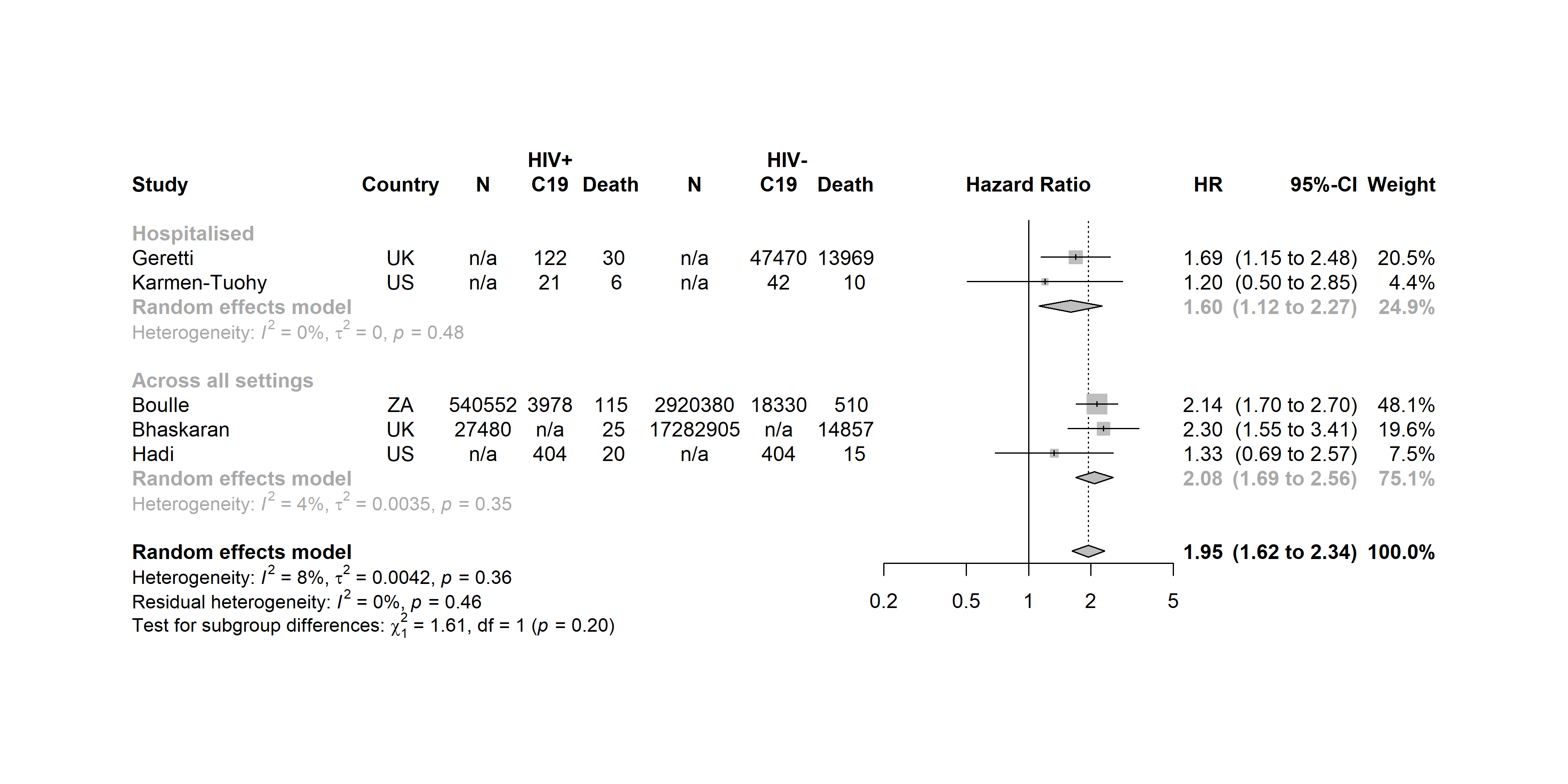


Notes: Country; UK = United Kingdom, US = United States of America, ZA = South Africa. HIV+ and HIV- refers to people with and without HIV. C19 refers to those with COVID19. The denominators (N) refer to the original population where reported: A cohort of people diagnosed with HIV and the wider general population without HIV. Not all studies reported this information. Results are reported as hazard ratios (HR) with 95% confidence intervals (95%CI).

# Figure S2 Meta-analysis of the effect of HIV on risk of COVID-19 death, by study design

#
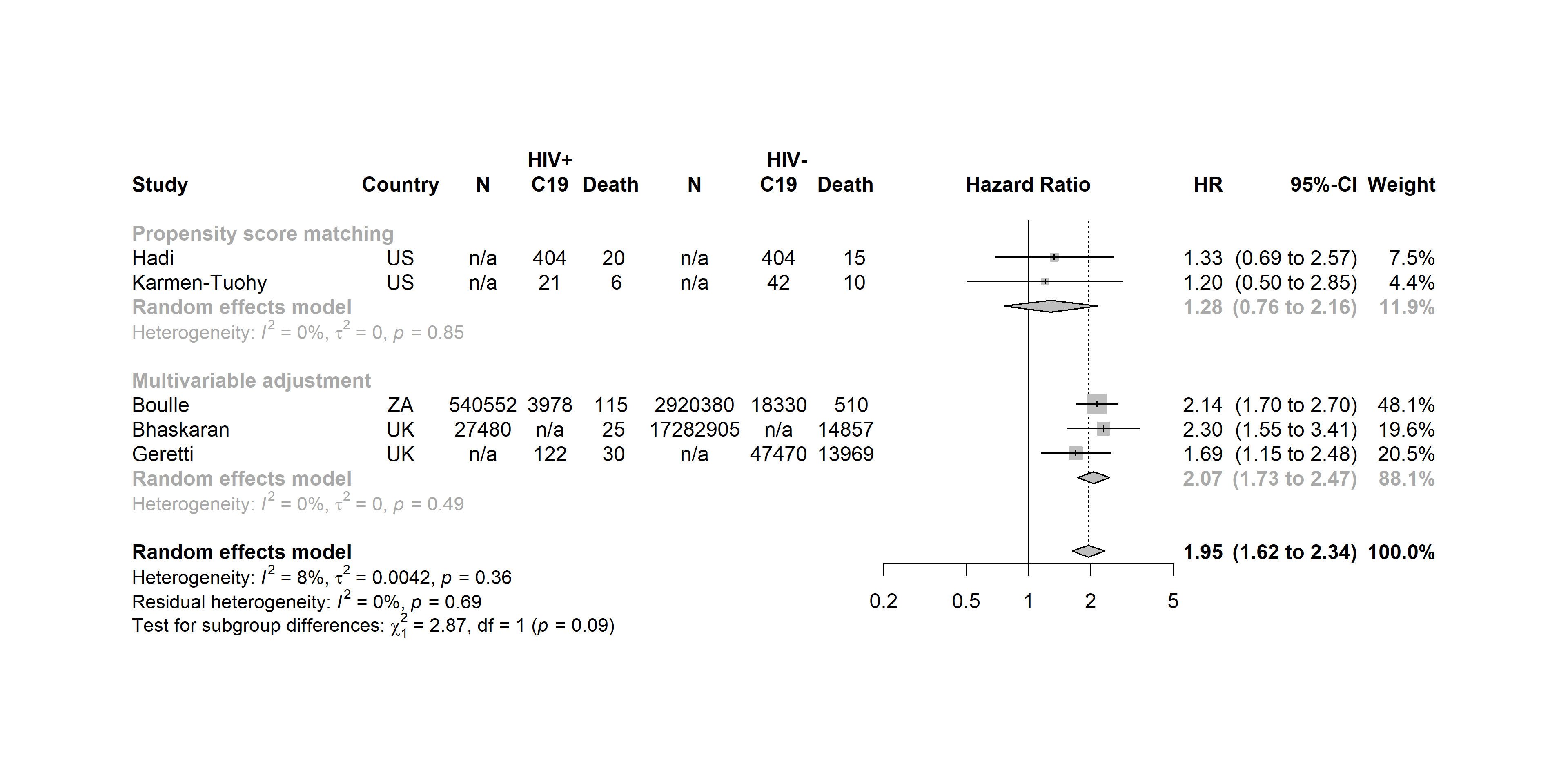


Notes: Country; UK = United Kingdom, US = United States of America, ZA = South Africa. HIV+ and HIV- refers to people with and without HIV. C19 refers to those with COVID19. The denominators (N) refer to the original population where reported: A cohort of people diagnosed with HIV and the wider general population without HIV. Not all studies reported this information. Results are reported as hazard ratios (HR) with 95% confidence intervals (95%CI).
